# Frequency of Genetic Testing Among Patients With Epithelial Ovarian, Fallopian Tube, and Peritoneal Cancers: A Strategy to Improve Compliance

**DOI:** 10.1155/ijog/9281891

**Published:** 2025-03-01

**Authors:** Jude Nawlo, Kevin Espino, Deanna Gerber, Meredith Akerman, Kent Chan, Edward Jimenez, Eva Chalas

**Affiliations:** ^1^Department of Gynecologic Oncology, New York University Grossman School of Medicine, New York, USA; ^2^Department of Gynecologic Oncology, New York University Grossman Long Island School of Medicine, Mineola, New York, USA; ^3^Department of Biostatistics, New York University School of Medicine, Mineola, New York, USA

## Abstract

**Purpose:** In 2014, the Society of Gynecologic Oncology (SGO) recommended universal germline testing for all patients with epithelial ovarian cancer (EOC), fallopian tube cancer (FTC), or peritoneal cancer (PC). Despite this position statement, genetic testing (GT) uptake among affected patients remains well below the universal testing goal. The aim of this study is to evaluate the impact of an internal policy change on the GT rate at a single institution.

**Patients and Methods:** This investigation was an Institutional Review Board (IRB)–approved (#22-00711) retrospective cohort study which took place at a single institution from June 2021 to April 2022. The study assessed GT uptake among patients diagnosed with EOC, FTC, and PC to evaluate the following internal policy change integrating point-of-care (POC) GT.

**Results:** A total of 272 patients were identified with 47 patients excluded due to nonepithelial tumors. Genetic counseling was documented in 94.2% of eligible patients (212/225) and completed in 90.2% (203/225). Of the 22 (9.8%) who were not genetically tested, 27% (6/22) were offered and declined. Deleterious mutations were identified in 22% (45/205) of patients tested. Of these, 82.2% (37/45) were in BRCA, 6.8% (3/45) in Lynch-associated mutations (MSH2, MSH6, MLH1, and PMS2), 4.4% (2/45) in RAD51, 4.4% (2/45) in BRIP1, and 2.2% (1/45) in an unknown deleterious mutation reportedly diagnosed at a different facility.

**Conclusion:** Internal policy developed based on analysis of compliance with the SGO position statement and subsequent implementation of POC testing led to a significant increase in GT, indicating improvement in quality medical care. GT rates in this population are markedly higher than reported in the literature.

## 1. Introduction

Genetic testing (GT) for patients with epithelial ovarian cancer (EOC), fallopian tube cancer (FTC), and primary peritoneal cancer (PC) remains below the universal testing goal despite recommendations by a number of national professional organizations [1–4]. The germline mutation rate in patients with EOC, FTC, ovarian cancer, and primary PC is consistently reported to be over 20%, underscoring the need for clinical evaluation [5–7]. Based on a compiled review of 35 studies conducted by Lin et al., the rate of genetic referral and GT among patients with ovarian cancer was 39% and 30%, respectively, without the integration of clinician-based interventions [2]. Specific factors, such as race and insurance status, were significant in impacting the frequency of GT, with Black and uninsured patients experiencing greater disparities.

GT offers both prognostic and predictive values through individualized therapy and facilitates cascade testing for at-risk family members, making it a critical component of clinical decision-making. Therefore, efforts to increase referral rates must be enhanced [8]. Significant advancements in molecular diagnostic testing for cancer predisposition genes have led to more comprehensive counseling and testing options for patients [9]. Current initiatives show that mainstreaming strategies, including referrals to genetics teams and oncologist-led efforts, substantially improve testing uptake and access in patients with EOC [10]. Supportive research reflects that physician-led, in-office GT accelerates testing timelines, expedites receipt of results, and helps overcome barriers in the underserved and uninsured populations [11]. Additionally, the integration of telemedicine encounters has contributed to reducing disparities in the utilization of GT services [12].

In the timeline of scientific progress, the understanding of hereditary breast and ovarian cancer (HBOC) syndrome evolved significantly in the late 20th century through initiatives like the Human Genome Project and the discovery of BRCA1/2 genes [13, 14]. These breakthroughs propelled advancements in the field of gynecologic oncology, particularly with the adoption of next-generation sequencing (NGS) technologies, the introduction of hereditary cancer risk panels, and the emergence of innovative treatment options like poly ADP-ribose polymerase (PARP) inhibitors [15–17]. GT is recommended for patients with EOC, FTC, and PC due to its prognostic implications on overall survival, making it the standard of care for this population [18]. Moreover, mainstream GT offered by nongenetics providers has been shown to increase testing uptake among these patients and significantly reduce healthcare costs [19].

**Figure 1:**
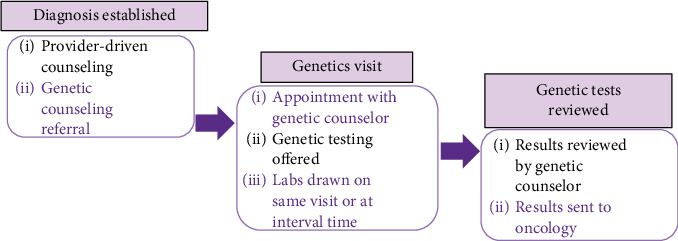
Former model of genetic testing (Gotimer et al. [20]).

This investigation was conducted as a follow-up retrospective cohort review of a quality improvement initiative led by Gotimer et al. The objective was to evaluate the impact of the Society of Gynecologic Oncology (SGO) statement endorsing universal GT uptake among patients with EOC, FTC, and PC [20]. In the original study by Gotimer et al., the GT process followed a stepwise approach in accordance with the SGO standard of care (Figure 1). After a cancer diagnosis, the oncology team referred the patient to a geneticist for consultation and testing. Once the results were obtained, the genetics team reviewed them with the patient and relayed the findings back to the oncology team. This framework often led to significant delays, with genetics consultations taking several months to up to a year, even requiring multiple reminders from the care team. Of note, this process is still widely implemented across the country and requires modification to enhance uptake.

The demand to improve GT uptake is further emphasized by its integration into clinical practice. Per Gotimer et al., 60% of patients with EOC, PC, and FTC underwent GT, and of those tested, 23% were found to have pathogenic mutations. In evaluating the impact of the SGO recommendation, Gotimer et al. observed that patients diagnosed after the SGO statement experienced reduced intervals from their first oncology visit to genetics consultation and from genetics referral to GT. These findings highlight the significant influence of the national recommendation on improving the efficiency of the GT process. Despite the positive impact of the SGO statement, the interval from referral to GT and initial consultation to GT still ranged up to 3 and 6 months, respectively, indicating opportunity for improvement. Although the model is aimed at expanding GT, it remained subject to delays due to heavy referral reliance and barriers involving consultation availability, among other factors.

The results of Gotimer et al. prompted an internal policy change to implement point-of-care (POC) GT during the office visit immediately following histologic confirmation of the cancer diagnosis. The hypothesis was that by offering GT at the time of diagnosis confirmation and performing the necessary laboratory work during the same visit, the GT rate would increase.

Preliminary findings from this study were presented as a conference abstract at the 2023 SGO Annual Meeting [21]. The abstract highlighted initial results on GT uptake following the internal policy change. This manuscript builds upon those findings by providing a detailed methodological framework, a comprehensive analysis of patient demographics and GT barriers, and an in-depth discussion of the broader implications for clinical practice and development.

## 2. Methods

A retrospective chart review was conducted to evaluate rates and outcomes of GT in the patient sample. The Institutional Review Board (IRB)–approved study (#22-00711) included patients diagnosed with primary ovarian cancer, FTC, and PC who received care at a single large, academic, nonprofit urban hospital in the period from June 2021 and April 2022. The study period was chosen to evaluate recent annual progress in GT. Descriptive statistics (mean ± standard deviation for age; frequency and percent for all categorical variables) were calculated. Analyses were performed using SAS Version 9.4 (SAS Institute Inc., Cary, NC). The gynecologic oncology practice where this study was conducted comprises five gynecologic oncologists with support by dedicated nursing staff and physician assistant providers who contribute to patient management and education. This multidisciplinary team plays a crucial role in ensuring comprehensive care and facilitates the integration of GT into routine practice.

**Figure 2:**
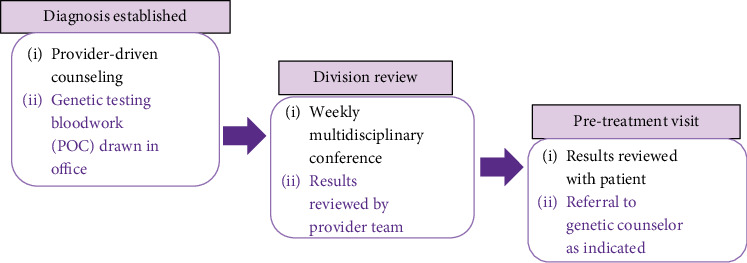
Internal policy change.

This internal policy change, adapted and modified based on results from Gotimer et al., introduced provider-initiated POC GT at the time of cancer diagnosis. Given the increasing nationwide demand on genetic support services, this approach is aimed at consolidating office visits and streamlining referrals by eliminating steps that would otherwise lead to patient care delays [22]. Using this model, patients with histologic confirmation of epithelial cancers were reviewed at a weekly interdisciplinary conference, and GT results were expediently obtained and evaluated. The results were then discussed with the patient at their pretreatment visit, and referral to genetic counseling was established if a pathogenic mutation was identified (Figure 2). GT was mainly carried out through companies such as Myriad, Invitae, and Ambry. Pathogenicity was determined based on the classifications provided by the respective GT companies.

## 3. Results

A total of 272 patients with confirmed diagnoses of primary ovarian cancer, FTC, and PC seen in outpatient visits were identified in the period of June 2021 and April 2022. After excluding patients without epithelial cancer diagnoses, the final sample size was 225 (*n* = 225). The average age of the cohort was 65 years, with 81% identifying as White, 7% as African American, 3% as South Asian, 3% as Asian, and 6% as other/unknown.

**Figure 3:**
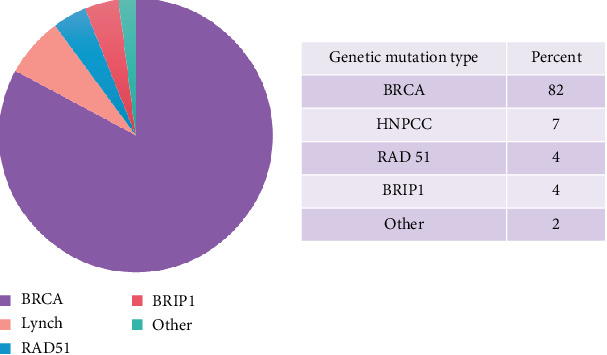
Rates of pathogenic mutations.

A total of 163 (72.4%) patients had EOC, 42 (18.7%) patients had FTC, and 20 (8.9%) patients had primary PC. GT counseling was documented in 94% of eligible patients (212/225). Following their diagnosis, 90% (203/225) completed GT, while 22 patients (9.8%) did not undergo GT as part of their routine cancer care. Mutations were identified in 22% of patients (45/205) who completed testing. Of those with mutations identified, 82.2% (37/45) were in BRCA1/2, 6.8% (3/45) in Lynch-related mutations, 4.4% (2/45) in RAD51, and 4.4% (2/45) in BRIP1 (see Figure 3). One patient (2.2%, 1/45) self-reported a pathogenic mutation from an outside facility prior to treatment at the study campus.

Twenty patients were found to have variants of unknown significance (VUS). Six (27%) of 22 patients did not undergo GT due to personal preference, and one patient (4%) died of disease prior to completion of GT. In 15 (68%) patients, the reasons for lack of testing were unknown.

## 4. Discussion

The data presented support the implementation of POC GT as an effective strategy to increase GT uptake among patients with EOC, FTC, and PC. In this cohort, where the risk of identifying a germline mutation is up to 25%, POC GT offers opportunity for expedited care and therapeutic benefits. Although POC testing has not yet been widely adopted within the framework of new diagnoses in oncology, studies have demonstrated its effectiveness in improving genetic counseling and GT uptake among patients newly diagnosed with breast and pancreatic cancer [23, 24]. The implementation of POC testing streamlines laboratory processes and ensures timely disclosure of results. This process requires strong interdisciplinary communication and planning, as well as systematic clinical workflow and infrastructure, to be most impactful.

**Figure 4:**
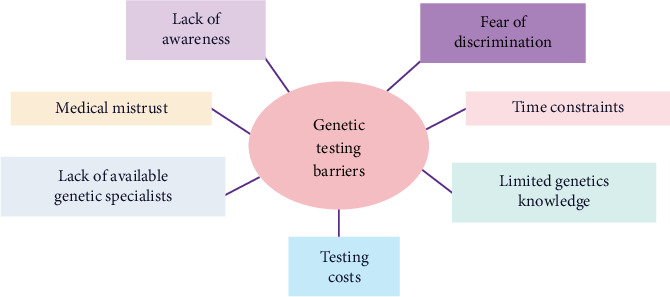
Barriers to genetic testing.

The incidence of pathogenic germline mutations in this cohort was comparable to nationally reported rates at approximately 20%. The GT compliance rate in this sample markedly exceeded that of the national average, which demonstrates the efficacy of the process and supports policy change affecting change in the clinical workflow. The standardized approach eliminates disparities associated with race and insurance status and optimizes efficiency of healthcare provision in this cohort. While the majority of the patients were insured under predominantly private and/or Medicare insurance policies, collaboration with GT companies provided access to financial aid. However, the association between insurance policy and GT for cancer predisposition genes remains a subject requiring study and is not all-encompassing of other deleterious mutations. A recent study revealed that health insurance guidelines may not be sufficient in differentiating whether there is a necessity to perform GT in specific cancer patients [25]. The reality of low uptake is a topic of national discourse, as patients without access to testing may suffer restricted treatment options, as well as blunted opportunity for cascade testing and disease prevention. Despite recommendations put forth by SGO, the nationwide GT rate in this patient population is only about 30% [26]. The poor uptake of GT underscores the significance in identifying and overcoming barriers that hinder universal GT. These barriers may include healthcare literacy limitations, financial burdens, and inconvenience with numerous clinical visits all in the midst of a new cancer diagnosis (see Figure 4) [27].

The strengths of this study include an institutional basis of comparison through Gotimer et al. in 2016. This investigation is one of the first to assess the role and impact of POC GT in the gynecologic oncology cohort, with the single-site standardized approach providing ease of implementation and consistency across the provider team. The limitations to consider include retrospective design with data collection via chart review and limitation to a single hospital campus, which may affect the generalizability to other hospital and clinic settings. Despite all this, the impressive uptake results observed with POC testing show incredible potential for standardizing this practice within the field of gynecologic oncology, decreasing the risk of disparity in offering the testing and favorably impacting the quality of healthcare for these patients and their relatives.

## 5. Conclusion

Internal policy change designed to integrate POC GT following histologic confirmation of ovarian cancer, FTC, and PC is effective in markedly increasing GT uptake in accordance with SGO recommendations. As the reported uptake rates remain low despite national input, this study documents opportunity for improvement in quality medical care. By implementing universal POC GT within this patient population, gynecologic oncology providers can facilitate more timely services and referrals, expand treatment options, and implement risk reduction strategies.

## Data Availability

The data used to support the findings of this study are available from the corresponding author and statistics team upon request.
